# Maximal Oxygen Consumption Is Reduced in Aquaporin-1 Knockout Mice

**DOI:** 10.3389/fphys.2016.00347

**Published:** 2016-08-10

**Authors:** Samer Al-Samir, Dominique Goossens, Jean-Pierre Cartron, Søren Nielsen, Frank Scherbarth, Stephan Steinlechner, Gerolf Gros, Volker Endeward

**Affiliations:** ^1^Vegetative Physiologie 4220, Abt. Molekular-und Zellphysiologie, Medizinische Hochschule HannoverHannover, Germany; ^2^Institut National de la Transfusion Sanguine–Institut National de la Santé et de la Recherche Médicale UMR_S1134Paris, France; ^3^Biomedicine, Department Health Science and Technology, Aalborg UniversityAalborg, Denmark; ^4^Institut für Zoologie, Tierärztliche Hochschule HannoverHannover, Germany

**Keywords:** aquaporin 1, Rhesus-associated glycoprotein, aquaporin 9, maximal oxygen consumption, knockout mice, arterial oxygen saturation, cardiac function of the heart of aquaporin-1-deficient mice

## Abstract

We have measured maximal oxygen consumption ($\dot{\text{V}}$_O2,max_) of mice lacking one or two of the established mouse red-cell CO_2_ channels AQP1, AQP9, and Rhag. We intended to study whether these proteins, by acting as channels for O_2_, determine O_2_ exchange in the lung and in the periphery. We found that $\dot{\text{V}}$_O2,max_ as determined by the Helox technique is reduced by ~16%, when AQP1 is knocked out, but not when AQP9 or Rhag are lacking. This figure holds for animals respiring normoxic as well as hypoxic gas mixtures. To see whether the reduction of $\dot{\text{V}}$_O2,max_ is due to impaired O_2_ uptake in the lung, we measured carotid arterial O_2_ saturation (S_O2_) by pulse oximetry. Neither under normoxic (inspiratory O_2_ 21%) nor under hypoxic conditions (11% O_2_) is there a difference in S_O2_ between AQP1_null_ and WT mice, suggesting that AQP1 is not critical for O_2_ uptake in the lung. The fact that the % reduction of $\dot{\text{V}}$_O2,max_ is identical in normoxia and hypoxia indicates moreover that the limitation of $\dot{\text{V}}$_O2,max_ is not due to an O_2_ diffusion problem, neither in the lung nor in the periphery. Instead, it appears likely that AQP1_null_ animals exhibit a reduced $\dot{\text{V}}$_O2,max_ due to the reduced wall thickness and muscle mass of the left ventricles of their hearts, as reported previously. We conclude that very likely the properties of the hearts of AQP1 knockout mice cause a reduced maximal cardiac output and thus cause a reduced $\dot{\text{V}}$_O2,max_, which constitutes a new phenotype of these mice.

## Introduction

Molecular dynamics simulations have shown that AQP1 and AQP4 may conduct CO_2_ and O_2_ through their central pore (Hub and de Groot, 2006; Wang et al., 2007; Wang and Tajkhorshid, 2010). This is likely to be also true for some other aquaporins such as AQP9. Experimental evidence for a physiologically meaningful conduction of CO_2_ by these three proteins has indeed been repeatedly reported (Endeward et al., 2006a; Musa-Aziz et al., 2009; Itel et al., 2012; Geyer et al., 2013). In addition both, molecular dynamics simulations and experimental evidence, have shown that Rhesus proteins conduct CO_2_ (Endeward et al., 2006a,b, 2008; Musa-Aziz et al., 2009; Hub et al., 2010). However, in our view clear experimental evidence for a physiologically relevant conduction of O_2_ does not exist for any of these gas channels.

It was our aim in this paper to study the role of these gas channels in systemic O_2_ transport by measuring maximal oxygen consumption in mice, which had one or two of these channels knocked out. We wanted to test, whether absence of one or two of these channels results in a diffusion problem that limits oxygen uptake. We chose AQP1_null_, Rhag_null_, and AQP9_null_ animals, because these three proteins occur in the red blood cell membrane of the mouse and because all three of them have been shown to act as CO_2_ channels either in human red cells (Endeward et al., 2006a,b, 2008) or in artificial expression systems (Musa-Aziz et al., 2009; Geyer et al., 2013). It should be appreciated that the principle of our measurement implies that not only lack of potential O_2_ channels in the red cell membrane, but also lack of such channels in lung tissue and in the tissues of peripheral organs, especially skeletal muscle, could be involved in a limitation of maximal oxygen consumption.

We propose from our data that lack of the three gas channels does not impair oxygen exchange in lung and tissues. However, lack of AQP1, which is associated with a significant reduction of maximal oxygen consumption, likely causes this effect via a reduced maximal cardiac output of the AQP1 knockout animals. In a preceding paper (Al-Samir et al., 2016) we have reported that the left ventricle of the hearts of AQP1-deficent mice has thinner walls than WT mice along with a reduced capillary density.

## Methods

### Animals

Breeding pairs of heterozygous AQP1 KO mice were kindly provided by Dr. Alan S. Verkman (San Francisco, USA; Ma et al., 1998). They were intercrossed to obtain homozygous AQP1 KO mice and WT littermate controls. AQP9 KO mice were those described by Rojek et al. (2007), Rhag_null_ mice were those described in Goossens et al. (2010). AQP1/Rhag double KO mice were obtained by intercrossing the two types of KO mice. All strains were occasionally intercrossed with Bl6 from the central animal facility of Hannover Medical School. For PCR genotyping of AQP1-knockout mice we used the DNA from tail snippets and specific the primers

AQP1 W: AAG TCA ACC TCT GCT CAG CTG GG
AQP1 Neo: CTC TAT GGC TTC TGA GGC GGA AAG
AQP1 KO: ACT CAG TGG CTA ACA ACA AAC AGG

in one single PCR reaction.

For PCR genotyping of AQP9-knockout mice with DNA isolated from tail snippets specific primers were used in one single PCR reaction:

F: 5′-AAC TGG GGA TAG TGG GAT TCA AAG A-3′ for AQP9 WT
R: 5′-GCC ACT AGC CAT GTG TTG GTA TTT C-3′ for AQP9 WT and KO
F: 5′-GTG CTA CTT CCA TTT GTC ACG TCC T-3′ for AQP9 KO.

For PCR genotyping of Rhag-knockout mice with DNA isolated from tail snippets specific primers were used in two separate runs:

RhAG WT-F: 5′-CCA TCT CTC CAG CCT AGC AAT TTT C-3′
RhAG WT-R: 5′-AAG TCA GGA AAG AGT CAT TGC ATG G-3
RhAG KO-F: 5′-TGC TAT GAC CAT TGG AAG CAT TGC-3′
RhAG KO-R: 5′-ATT GCA TCG CAT TGT CTG AGT AGG-3′

The absence of the proteins AQP1, AQP9, and Rhag, respectively, has been demonstrated in the single KO mice by Ma et al. (1998), Rojek et al. (2007), and by Goossens et al. (2010).

The mice used in this study had an average weight of 26–31 g and comprised about equal numbers of males and females. For the measurements of resting and maximal oxygen consumption, the groups were weight-matched. Animal experiments were approved by the Niedersächsisches Landesamt für Verbraucherschutz und Lebensmittelsicherheit (No. 33.12-42502-04-12/0973).

### Measurement of animal oxygen consumption

Measurements of animal oxygen consumption were done on conscious mice. Oxygen consumption $\dot{\text{V}}$_O2_ was measured under two conditions, firstly under “resting” conditions using air at room temperature as the inspiratory gas ($\dot{\text{V}}$_O2, rest_), secondly under conditions maximizing $\dot{\text{V}}$_O2_ by using either a (normoxic) mixture of 79% He with 21% O_2_ (Helox) cooled down to 4°C, yielding normoxic $\dot{\text{V}}$_O2,max_, or a (hypoxic) mixture of 79% He with 11% O_2_ and 10% N_2_ at 4°C and yielding hypoxic $\dot{\text{V}}$_O2,max_. While the measurement using air represents the classical open system for $\dot{\text{V}}$_O2_ determination, which measures gas flow together with inflowing and outflowing O_2_ concentrations, the measurement using He/O_2_ follows the same principle but maximizes $\dot{\text{V}}$_O2_ of the animals by withdrawing heat from their lungs. The increased heat loss is caused by the employed gas mixture of 79% He and O_2_, which conducts heat 4 times more effectively than air (Rosenmann and Morrison, 1974). This heat loss causes a drastic increase in $\dot{\text{V}}$_O2_, initiated by the thermoregulatory system aiming to maintain core body temperature. Comparisons with protocols for maximal physical exercise and other techniques have shown that this elevated $\dot{\text{V}}$_O2_ indeed represents the maximal oxygen consumption achieved by the animals, $\dot{\text{V}}$_O2,max_, which has a value 5–7 times higher than the basal $\dot{\text{V}}$_O2_ of mice of ~0.03 ml O_2_/g body weight/min (Segrem and Hart, 1967; Rosenmann and Morrison, 1974; Chappell, 1984; von Engelhardt et al., 2015).

**Figure 1:**
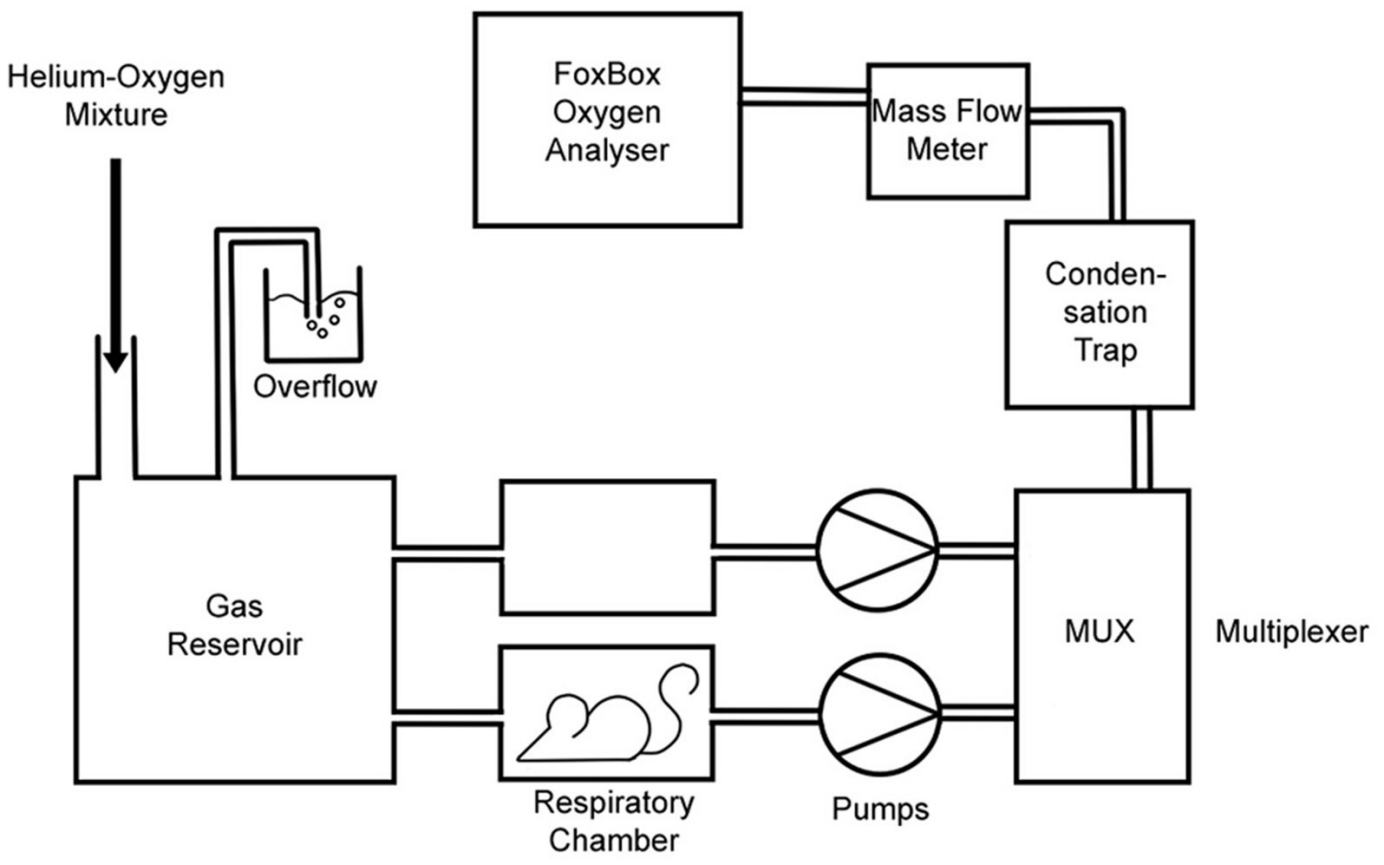
**Experimental set-up for measurement of maximal oxygen consumption**. Pumps draw gas from the gas reservoir through the respiratory chambers and from there via the multiplexer through a water condensation trap and through the flow meter into the oxygen analyzer. For $\dot{\text{V}}$_O2_, max determination, the gas reservoir contains 21% oxygen/rest He, and the gas reservoir and respiratory chambers are placed into a cold room at 4°C. For resting $\dot{\text{V}}$O2 determination, the gas reservoir contains air, and the entire set-up is placed in room air at 21°C. The empty chamber serves as a control of the inspiratory gas. Usually a third respiratory box was mounted in parallel to the two boxes shown, containing another animal. In this case, the three boxes contained a WT mouse, a KO mouse and no animal, respectively. The gas coming from the empty box was used as a reference gas indicating the O_2_ concentration in the gas reservoir.

The basic experimental setup for both types of measurement is illustrated in Figure 1. The setup shown allowed us to measure $\dot{\text{V}}$_O2_ of one mouse that was placed into one of the two respiratory chambers shown. The second chamber was used to obtain a reference gas value. Respiratory chamber dimensions were 15.5 × 8.5 × 10.5 cm. The two pumps drew gas from the gas reservoir at a constant flow rate of 35 l/h through each of the chambers, one with and one without a mouse. The valves of the flow multiplexer (MUX, Sable Systems, North Las Vegas, NV 89032 USA) were controlled by a PC. This provided values of flow rates (MKS Instruments, Munich, Germany; Model: 035CC-01000SVS008) and oxygen concentrations of the outflowing gases (FoxBox; Field Oxygen Analysis System; Sable Systems, North Las Vegas, NV 89032 USA), alternating every 90 s between the two boxes. The gas coming from the empty chamber served as a reference gas for the O_2_ concentration of air or the He-O_2_ mixture in the reservoir, respectively, i.e., of the inspiratory gas. The flow meter was calibrated by flushing it at known flow rates either with air or with the suitable helium mixture. $\dot{\text{V}}$_O2_-values were determined from the difference in O_2_ concentrations between the gases leaving the two chambers, multiplied by the flow rate of the gas leaving the chamber occupied by the mouse. $\dot{\text{V}}$_O2_ was normalized for standard (STPD) conditions (0°C, dry).

For simultaneous determination of $\dot{\text{V}}$_O2_ in two mice, a third respiratory box was arranged in parallel to the two boxes indicated in the figure. Then, the flow multiplexer alternated between the three boxes and the gas coming from the empty chamber again served as a reference gas. The same gas flow was sucked continuously from the reservoir through all three chambers. Usually, $\dot{\text{V}}$_O2_ was measured in parallel for one KO and one WT mouse of similar body weight. Measuring periods for $\dot{\text{V}}$_O2_ determinations were 45 min, but in the $\dot{\text{V}}$_O2,max_ measurements the maximum was usually reached after a few minutes. It should be noted that the measurements with room air at 21°C were carried out substantially below the thermoneutrality temperature of mice (28°C; Hoevenaars et al., 2014).

### *In vivo* measurement of arterial oxygen saturation and heart and respiratory rate

Carotid artery oxygen (S_O2_) saturations together with heart rates (HR) and respiratory rates (RR) were measured *in vivo* with a MouseOX Plus pulse oxymeter (Starr Life Sciences Corp., Oakmont, PA 15139 USA) using ThroatClip sensors size M or S depending on animal size. Data were recorded with the MousOX Plus premium monitoring software. Neck and throat of the animals where shaved to ensure optimal contact of the sensors. The carotid S_O2_ measurements were performed only on animals respiring 79% He with 21 or 11% O_2_ at 4°C, using the same setup as employed for $\dot{\text{V}}$_O2,max_ experiments (Figure 1). S_O2_, HR, and RR were recorded continuously for 20 min for each animal studied. Due to the greater agility of the animals respiring air at 21°C, the sensor did not remain in a stable position, therefore S_O2_ measurements under resting conditions could not be performed reliably.

### Statistical treatment

The results of $\dot{\text{V}}$_O2,max_ and S_O2_ showed without exception normal distribution when tested according to D'Agostino & Pearson. All pairs of data from WT vs. KO animals were subjected to Student's *t*-test (unpaired). The software used for both tests was GraphPad Prism 6.01 (GraphPad Software, Inc., La Jolla, California, USA). Statistical significances were calculated for each pair of WT/KO measurements. *N*-values given in the tables refer to number of animals studied.

## Results

### Resting oxygen consumption of conscious mice

Specific resting oxygen consumption of WT mice as measured under exposure of the animals to air at 21°C varies between 0.066 and 0.072 ml O_2_/g body weight/min for the different mouse strains listed in Table 1 (2nd column). These values, as already discussed above, are about twice the expected basal $\dot{\text{V}}$_O2_-value for mice of ~0.03 ml/g/min (Segrem and Hart, 1967; Rosenmann and Morrison, 1974; von Engelhardt et al., 2015). The higher $\dot{\text{V}}$_O2_ may be caused by two factors: (1) the temperature of the measurement, 21°C, is markedly below thermoneutrality, which causes an enhanced metabolism, and (2) the tendency of the animals to exhibit low to moderate physical activity by moving around in their cages (Figure 1). Comparing the resting $\dot{\text{V}}$_O2_-values of the four mouse strains, it is seen that in two cases, Rhag KO and AQP9 KO, resting $\dot{\text{V}}$_O2_ is not significantly different between KO and WT, but in the other two cases, AQP1 KO and AQP1-Rhag double KO, $\dot{\text{V}}$_O2_ is significantly lower in KO mice compared to WT mice. This seems to point to a special property of the AQP1-KO strain.

**Table 1:** **Results of $\dot{\text{V}}$_O2_ measurements in KO and WT mice (in ml O_2_/g/min)**.

	**Normoxia**		**Hypoxia**	**Body weight**
	**$\dot{\text{V}}$_O2, rest_(air)**	**$\dot{\text{V}}$_O2, max_ (Helox)**	**$\dot{\text{V}}$_O2, max_ (He, 11%O^2^)**	
AQP1 WT	0.066 ± 0.011 (13)	0.165 ± 0.016 (13)	0.100 ± 0.013 (12)	26.7/27.9 g
AQP1 KO	0.053 ± 0.009^p2^ (12)	0.142 ± 0.053^p1^(12)	0.087 ± 0.009^p3^ (12)	25.6/25.9 g
Rhag WT	0.071 ± 0.025 (8)	0.132 ± 0.016 (8)	0.081 ± 0.015 (8)	28.4/28.6 g
Rhag KO	0.077 ± 0.013^ns^ (7)	0.142 ± 0.010^ns^ (7)	0.086 ± 0.020^ns^ (7)	28.6/28.9 g
AQP1-Rhag WT	0.068 ± 0.012 (15)	0.151 ± 0.025 (15)	0.093 ± 0.015 (15)	26.7/27.4 g
AQP1-Rhag DKO	0.050 ± 0.012^p5^ (14)	0.130 ± 0.026^p4^(14)	0.082 ± 0.017^ns^ (14)	26.8/26.2 g
AQP9 WT	0.072 ± 0.018 (13)	0.137 ± 0.014 (13)	0.090 ± 0.013(13)	30.7/30.0 g
AQP9 KO	0.063 ± 0.018^ns^ (14)	0.133 ± 0.018^ns^(14)	0.090 ± 0.014^ns^ (14)	30.8/31.5 g

$\dot{\text{V}}$_O2, rest_ are measurements of mice at rest in air at 20–24°C, $\dot{\text{V}}$_O2,max_ are measurements of maximal oxygen consumption in the presence of 79% He in the inspiratory gas. Means are given together with SD. Numbers in brackets are number of animals studied. Body weights give on the left numbers for normoxia, on the right for hypoxia. Levels of significance are given for KO- vs. WT-values: ns = not significantly different, p1 = 0.002, p2 = 0.02, p3 = 0.01, p4 = 0.03, p5 = 0.004. Each comparison is between one set of measurements with WT and the corresponding one with KO (DKO) animals.

### Maximal oxygen consumption of conscious mice

As described in Methods, the principle employed here to obtain $\dot{\text{V}}$_O2,max_ is to withdraw heat from the animals via their lung. This is achieved by exposing the animals not only to an environment of 4°C but in addition letting them respire a gas mixture containing 79% Helium (plus either 21 or 11% O_2_), which is also cooled down to 4°C. The excellent heat conduction by He greatly accelerates heat loss and induces an increase in the animals' $\dot{\text{V}}$_O2_ to the maximum. For normoxic conditions, the 3rd column of Table 1 shows the results for $\dot{\text{V}}$_O2,max_ as obtained by inspiration of Helox at 4°C. It is apparent that only in the case of AQP1-deficient mice, AQP1-KO and AQP1-Rhag double KO, the $\dot{\text{V}}$_O2,max_-value is markedly and significantly lower in KO than in WT mice, with differences of 16% in both animal strains. Neither Rhag-KO nor AQP9-KO show a difference to WT. We conclude that lack of AQP1 significantly reduces the maximal oxygen consumption under normoxic conditions. Compared to their basal $\dot{\text{V}}$_O2_ of ~0.03 ml O_2_/g/min, the WT animals exhibit a 4.5–5.5 times elevated $\dot{\text{V}}$_O2,max_, which is in excellent agreement with the literature (Segrem and Hart, 1967; Rosenmann and Morrison, 1974; Lechner, 1978; Chappell, 1984).

Under hypoxic conditions, $\dot{\text{V}}$_O2,max_ is in general drastically reduced to 60–65% of the normoxic values due to the hypoxia induced by 11% O_2_ in the inspired gas. A significant difference between KO- and WT-values is only seen in the case of the AQP1-KO mouse, where $\dot{\text{V}}$_O2,max_ is 15% lower in KO than in WT mice. A similar difference in AQP1-Rhag double-KO mice does not reach statistical significance. In Rhag-KO and AQP9-KO mice there is no difference compared to the respective WT animals. We conclude that, under hypoxia, lack of AQP1 causes a very similar reduction in $\dot{\text{V}}$_O2,max_ as we see it in normoxia. The two other gas channels are without effect.

### Heart rates and carotid artery oxygen saturations of conscious mice under conditions of maximal oxygen consumption

Table 2 gives the results of the pulse oximetric determinations of carotid arterial S_O2_, HR, and RR. In normoxia (2nd and 3rd columns), S_O2_ has a normal value of 97% both in WT and KO mice. Heart rates are greater than 700 min^−1^, indicating a maximally increased cardiac activity under Helox respiration (Hoit and Walsh, 2002, p. 284; Segrem and Hart, 1967; Kramer et al., 1993; Desai et al., 1997; Schuler et al., 2010, and personal communication by Beat Schuler). There is no significant difference between HR of KO and WT mice. The same holds for the RR-values.

**Table 2:** **Results of pulse oximetry in AQP1-KO and WT mice**.

	**AQP1-WT Normoxia (*N* = 8)**	**AQP1-KO Normoxia (*N* = 6)**	**AQP1-WT Hypoxia (*N* = 9)**	**AQP1-KO Hypoxia (*N* = 7)**
S_O2_,a (%) [at $\dot{\text{V}}$_O2_,max]	96.8 ± 1.1	96.9 ± 1.1^ns^	64.2 ± 3.1	65.5 ± 2.2^ns^
HR (min^−1^) [at $\dot{\text{V}}$_O2_,max]	770 ± 7	717 ± 28^ns^	736 ± 11	677 ± 31^ns^
RR (min^−1^) [at $\dot{\text{V}}$_O2_, max]	192 ± 13	168 ± 10^ns^	197 ± 13	183 ± 20^ns^

S_O2_, a arterial oxygen saturation (taken above the carotid artery), HR heart rate, RR respiratory rate. All measurements were obtained in about the same time interval as the $\dot{\text{V}}$_O2_, max measurements, a few minutes after the start of exposure to a helium mixture (21% O_2_, 79% He in normoxia, 11% O_2_, 10% N_2_, 79% He in hypoxia). Given are means ± SD. Statistical significances refer to comparisons of pairs of sets of KO- vs. WT-values (unpaired t-test). Levels of significance are from top down 0.92, 0.12, and 0.17 (3rd vs. 2nd column) and 0.73, 0.11 and 0.58 (5th vs. 4th column). Mean body weights were between 25 and 31 g.

The 4th and 5th columns of Table 2 give the oximetric results under hypoxia. Arterial S_O2_ is markedly reduced to about 65%, but there is again no difference between KO and WT. Likewise, values of HR and RR are not different between KO and WT; they are also not different from the values under normoxia. In conclusion, AQP1 deficiency does not seem to affect arterial oxygen saturation and heart as well as respiratory rate.

## Discussion

### Can the hypoxic reduction of $\dot{\text{V}}$_O2,max_ in wildtype animals under hypoxia be fully explained by the reduced arterial S_O2_?

Since all WT animals can be assumed to have normal cardiac function, it seems likely that the drastically reduced $\dot{\text{V}}$_O2,max_ seen under hypoxia is solely due to the markedly reduced arterial S_O2_ observed in this condition (Table 2). We discuss in this paragraph, whether this assumption is plausible in view of the existing literature. Judged from the heart rates of the WT animals seen in Table 2, it appears that cardiac activation is close to maximal and identical under normoxia and hypoxia. If indeed cardiac output is identical for WT under normoxia and hypoxia, the reduction of O_2_ consumption must be exclusively due to a reduced oxygen extraction form arterial blood under hypoxia. On average, WT animals under hypoxia (Table 1) show a $\dot{\text{V}}$_O2,max_ amounting to 63% of $\dot{\text{V}}$_O2,max_ under normoxia. From the arterial O_2_ saturations given in Table 2 for WT under normoxia and hypoxia, it can be estimated that the diminished O_2_ consumption under hypoxia is fully explicable by the diminished arterial O_2_ saturation in hypoxia: if mixed venous S_O2_ is taken to be about 10% in both normoxic and hypoxic animals, arterio-venous S_O2_ differences of 87% in normoxia and of 55% in hypoxia are calculated. Indeed, 55 turns out to be 63% of 87, indicating that the difference in O_2_ extraction fully explains the difference in $\dot{\text{V}}$_O2,max_. A number of 10% for mixed venous S_O2_ is realistic in view of measurements performed under maximal aerobic exercise in normoxia and hypoxia in goats, which yielded mixed venous S_O2_-values between 9 and 4.4% (Crocker and Jones, 2014). Although it cannot be excluded that in the present measurements mixed venous S_O2_ is somewhat different in normoxia and hypoxia, a deviation of, say, 5% from the assumed venous S_O2_ would only slightly affect this estimate. We conclude that the reduced $\dot{\text{V}}$_O2,max_ can be satisfactorily explained by the diminished arterial S_O2_ together with a reasonable value for mixed venous S_O2_.

### Lack of AQP1, but not of Rhag and AQP9, reduces $\dot{\text{V}}$_O2,max_—possible mechanisms

Table 1 shows that lack of AQP1 reduces $\dot{\text{V}}$_O2,max_ by about 15%, under both normoxia and hypoxia. This constitutes a new phenotype of AQP1-deficient mice, and might be caused by either reduced cardiac output or impaired gas exchange in AQP1-deficient mice. Although in our view there is no clear experimental evidence for a role of AQP1 in O_2_ transport across membranes, it has been shown by molecular dynamics simulations that AQP1 constitutes a channel for O_2_ as it does for CO_2_ (Wang et al., 2007). This fact has been invoked to explain experimental data on the interrelationship between AQP1 and HIF (hypoxia inducible factor) expression (Echevarría et al., 2007).

AQP1 is almost universally present in the capillaries of the body, notably in the lung, the heart and skeletal muscle (Nielsen et al., 1993; Rutkovskiy et al., 2013). It is also present in the red blood cell membrane, where is has been shown to act as a CO_2_ channel (Endeward et al., 2006a). If AQP1 contributed to O_2_ exchange in red blood cells, in the lung and in the periphery, it would be conceivable that lack of AQP1 is associated with retarded O_2_ diffusion in the lung as well as in the periphery. We have shown previously (Al-Samir et al., 2016) that a diffusion problem does not exist in the anesthetized AQP1-deficient mouse under conditions of a low rate of O_2_ consumption, neither in the lung nor in the periphery. The likelihood that a diffusion problem becomes apparent, however, increases with increasing O_2_ flow, i.e., O_2_ consumption, and with decreasing gradients of pO_2_ across the lung and/or peripheral diffusion barriers. This means, a diffusion limitation is most likely to show up when (a) $\dot{\text{V}}$_O2_ is maximal and (b) the animal inspires a hypoxic gas mixture. Therefore, we have measured in this study $\dot{\text{V}}$_O2_ under conditions maximizing the animals' metabolism and under hypoxia.

In view of recent evidence on the morphological and functional properties of the hearts of AQP1-deficient mice (Montiel et al., 2014; Al-Samir et al., 2016), we propose here that the reduced maximal $\dot{\text{V}}$_O2_ of these mice (Table 1) is due to a reduced maximal cardiac output. In the following, we will discuss the available evidence for impaired gas exchange vs. reduced cardiac output.

#### Effect of hypoxia on $\dot{\text{V}}$_O2,max_ argues against a diffusion problem

It is characteristic for a limitation by diffusion that the limitation is aggravated when the O_2_ partial pressure (pO_2_) gradient that drives the diffusion process is reduced. This happens in the lung as well as in the periphery when the animals are exposed to hypoxia. Our results do not show such a limitation. Under hypoxia, when alveolar pO_2_ is cut down to roughly one half, the relative reductions of $\dot{\text{V}}$_O2,max_ (Table 1) caused by the lack of AQP1 (15%, 13%) are identical to those observed under normoxia (16%, 16%). This clearly argues against an O_2_ diffusion problem due to the lack of AQP1. This argument applies to pulmonary as well as peripheral gas exchange.

#### No limitation of pulmonary O_2_ exchange apparent from arterial O_2_ saturation

For pulmonary gas exchange, this conclusion is supported most directly by the data of Table 2. The arterial oxygen saturations obtained by pulse oximetry during conditions of $\dot{\text{V}}$_O2,max_ show no difference between WT and KO animals, both in normoxia and hypoxia. Thus, even under conditions of maximal challenge of O_2_ diffusion by $\dot{\text{V}}$_O2,max_ in combination with hypoxia, the blood passing though the lung is equally (and fully) equilibrated with alveolar pO_2_ in KO as well as in WT mice. These data rule out a limiting role of O_2_ diffusion in the lung. It will be desirable to confirm in the future the normal diffusion properties of the lungs of AQP1-KO mice by directly measuring their diffusion capacities (Fallica et al., 2011). As just discussed, our data also argue against a diffusion limitation of peripheral O_2_ supply, although direct evidence for this would have required knowledge of venous O_2_ saturation.

The conclusion regarding the absence of diffusion limitation in the periphery is in line with the distribution of AQP1 in the lung and the periphery. Both in the lung and in skeletal muscle, the exclusive or dominant site of expression is the capillary endothelium. Thus, if lack of AQP1 causes no diffusion problem in the lung, where AQP1 normally is most prominently expressed (Nielsen et al., 1993), it is not likely that it will cause a problem in heart and skeletal muscle.

#### Comparison of resting vs. maximal $\dot{\text{V}}$_O2_

As explained above, increasing $\dot{\text{V}}$_O2_ can—like exposure to hypoxia—uncover a diffusion limitation. Thus, we ask, whether in AQP1-deficient mice a reduction in $\dot{\text{V}}$_O2,max_ is seen (Table 1, 3rd column, lines 1 and 2, and lines 5 and 6) that is not seen in $\dot{\text{V}}$_O2, rest_ (Table 1, 2nd column, lines 1 and 2, and lines 5 and 6). The data given here suggest that a reduction of $\dot{\text{V}}$_O2_ is present under resting conditions as well as under conditions of maximal O_2_ consumption. Thus, no diffusion limitation would become apparent here. However, the finding just described is in clear contradiction to own previous observations obtained with the same mice under anesthesia (Al-Samir et al., 2016). The $\dot{\text{V}}$_O2_-values of WT mice observed in this preceding study were identical to those given in Table 2 for resting conditions. However, there was no difference in $\dot{\text{V}}$_O2, rest_ between WT and KO mice in this study. We believe it likely therefore that the conscious AQP1-deficient mice of the present study exhibit a lower $\dot{\text{V}}$_O2, rest_ than WT animals because of the known reduced spontaneous physical activity of AQP1-deficient mice (Boron, 2010). Accordingly, basal $\dot{\text{V}}$_O2_ of AQP-KO animals is likely to be equal to that of WT animals, but $\dot{\text{V}}$_O2,max_ is reduced in KO compared to WT animals. We consider it likely, nevertheless, that this does not reflect a diffusion limitation but is caused by the properties of the hearts of WT and KO animals, as explained below.

#### Role of red cell membranes as barriers to O_2_ diffusion

If AQP1, which is present in mouse as well as in human red cells, would contribute to O_2_ transfer across the membrane, the presence or absence of AQP1 could make a difference in pulmonary as well as peripheral gas exchange. As discussed above, the present results suggest that this is not the case. While no measurements of the O_2_ permeability of mouse or human red cells are available, such measurements have been reported for CO_2_. Under the assumption that AQP1 is a channel for O_2_ as it is for CO_2_ (Wang et al., 2007), the permeabilities measured for CO_2_ might be similar for O_2_. While AQP1 makes a major difference for the CO_2_ permeability of human red cells (Endeward et al., 2006a,b) as does RhAG (Endeward et al., 2008), this is not so clear for mouse red cells. Yang et al. (2000) and Ripoche et al. (2006) found no difference in CO_2_ permeability of the red cells of mice either lacking AQP1 or Rhag. Own unpublished measurements support this (T. Meine, S. Al-Samir, V. Endeward, G. Gros). Thus, it seems possible that the red cell membranes of the knockout mice have identical properties to those of WT, and thus a differential contribution of KO and WT red cells to peripheral and pulmonary gas exchange would indeed not be expected.

#### Can the heart of the AQP1-deficient mouse be responsible for reduced $\dot{\text{V}}$_O2,max_?

If we reject an interpretation of the present data in terms of a diffusion problem, we are left with the known reduced left ventricular weight and wall thickness of the KO animals (Montiel et al., 2014; Al-Samir et al., 2016). This must be expected to be associated with a reduced maximal cardiac output and thus reduced $\dot{\text{V}}$_O2,max_. Since in the preceding study we have shown that cardiac function of AQP1-deficient animals under resting conditions is entirely normal including stroke volume and heart rate, this interpretation nicely explains why (normoxic) resting $\dot{\text{V}}$_O2_ under anesthesia is not reduced (cf. Al-Samir et al., 2016), but (normoxic) $\dot{\text{V}}$_O2,max_ is impaired (Table 1, 3rd column), as just discussed. It likewise explains satisfactorily why $\dot{\text{V}}$_O2,max_ is reduced to the same extent in normoxia and hypoxia, as discussed above.

We conclude that several lines of evidence argue against the existence of an O_2_ diffusion problem in mice lacking AQP1. This does not rule out that AQP1 acts as a pore for O_2_, but can be due to a rather high background permeability of biological membranes without AQP1. This would be in agreement with the very high O_2_ permeabilities observed by Subczynski et al. (1989, 1992) in artificial phospholipid and in biological membranes. If the basal O_2_ permeability of these membranes is so high, O_2_ transfer across AQP1 would not noticeably increase O_2_ permeation across the membrane. This is quite in contrast to what we and others have shown for the case of CO_2_ (Endeward et al., 2006a, 2008; Musa-Aziz et al., 2009; Itel et al., 2012), where AQP1 generates a significant increase in CO_2_ permeability of membranes containing a major fraction of cholesterol. Instead, AQP1 deficiency obviously limits maximal oxygen consumption by being associated with a reduced cardiac muscle mass and wall thickness, which must be postulated to go along with a reduced maximal cardiac output.

## Author contributions

Concept of study: SA, SS, GG, VE. Experiments: SA, FS, VE. Interpretation of data: SA, SS, GG, VE. Contribution of materials and animals: DG, JPC, SN. Writing the draft of the paper: GG. Contribution to final version of the paper: SA, DG, JPC, SN, FS, SS, GG, VE.

## Funding

We thank the Deutsche Forschungsgemeinschaft for funding this work with grant No. EN 908/2-1.

### Conflict of interest statement

The authors declare that the research was conducted in the absence of any commercial or financial relationships that could be construed as a potential conflict of interest.
